# Editorial: Obesity and cancer: the possible molecular links

**DOI:** 10.3389/fcell.2025.1542429

**Published:** 2025-02-11

**Authors:** Biswarup Basu, Debanjan Chakroborty, Chandrani Sarkar

**Affiliations:** ^1^ Department of Neuroendocrinology and Experimental Hematology, Chittaranjan National Cancer Institute, Kolkata, India; ^2^ Department of Pathology, University of South Alabama, Mobile, AL, United States; ^3^ Cancer Biology Program, Mitchell Cancer Institute, University of South Alabama, Mobile, AL, United States; ^4^ Department of Biochemistry and Molecular Biology, University of South Alabama, Mobile, AL, United States

**Keywords:** obesity, breast cancer, lymphoma, prostate cancer, comorbidities

Obesity is a global public health issue that adversely affects several disease pathogenesis and prognosis, including cancer, by increasing the risk and the number of deaths associated with cancer (Wang et al., 2019). The underlying molecular mechanisms of obesity-associated cancer progression are unclear. With worldwide growing obesity, the obese tumor microenvironment, in addition to cancer patients’ socio-economical or ethnic perspectives, has emerged as a new area for understanding the dynamics of cancer initiation and outcomes obtained and for developing improved approaches to cancer management. The Research Topic “Obesity and Cancer: The Possible Molecular Links” was developed to highlight novel, original research findings, and critical reviews focusing on identifying the link between obesity and cancers. With three original research studies and one review article, this Research Topic highlights the interconnecting issues regulating obesity and cancer progression. Furthermore, the Research Topic has included epidemiological findings that reinforce the role of obesity in cancer progression by assessing the effect of neighborhood obesogenic environment on cancer risk and mortalities.

Obese breast cancer (BCa) patients, particularly postmenopausal women, have an increased risk of hormone receptor (HR)-positive BCa compared to lean women and often demonstrate more aggressive forms of the disease and face numerous challenges during therapy (LeVee and Mortimer, 2023). Though in premenopausal women, obesity is reported to reduce the risk of BCa, after BCa diagnosis, obesity worsens overall survival (OS) in all BCa subtypes (LeVee and Mortimer, 2023). The interplay between adipocytes and tumor cells contributing to the adipose microenvironment significantly influences tumor growth and response to therapy (Nieman et al., 2013). Kakkat et al. reviewed the complex relationship between BCa cells and adipocytes during tumor development, progression, and therapeutic response. The review discusses how circulating adipokines influence BCa progression. While adipokines like leptin, resistin, chemerin, visfatin, osteopontin, apelin, and lipocalin 2 promote BCa pathogenesis, adiponectin confers a protective effect by suppressing breast carcinogenesis (Nehme et al., 2022). As leptin exerts an opposite effect by promoting BCa initiation, growth, and metastasis, the adiponectin-leptin ratio plays a critical role in breast tumorigenesis (Grossmann and Cleary, 2012). Cancer-associated adipocytes (CAAs) secrete factors influencing immune cell recruitment, functions, differentiation, and immune escape, thereby promoting tumor progression. Adipocytes also contribute to the formation of extracellular matrix (ECM), which in turn promotes metastatic dissemination and cancer advancement. Furthermore, adipocytes stimulate resistance to therapy by activating multiple signaling pathways that promote angiogenesis, increase tumor cell proliferation, and decrease apoptosis. A thorough investigation of the effects of strategies in weight loss on BCa progression and therapeutic response and the molecular pathways affected is essential for the development of novel therapeutic approaches for BCa patients.

In their original research article, Wu et al. studied the impact of body mass index (BMI) on malignant lymphoma. Numerous prior reports indicate a positive link between high BMI and both B cell derived Hodgkin’s lymphoma (HL) and non-Hodgkin’s lymphoma (NHL), derived from B cells and T cells, indicating that obesity is a potential risk towards malignant lymphoma (Larsson and Wolk, 2011; Murphy et al., 2013). Interestingly, some studies also indicate that obese or overweight patients have a better favorable prognosis in malignant lymphoma, making the role of obesity in lymphoma controversial (Landgren et al., 2005; Ho et al., 2014). In their study, Wu et al. determined the potential link between malignant lymphoma and BMI through two-sample Mendelian randomization. The study took into account 369 cases of HL, 209 cases of diffused large B-cell lymphoma, 522 cases of follicular lymphoma, 150 cases of mature T/NK- cell lymphomas, and 533 cases of other and unspecified types of NHL. The study findings indicate that adipose tissue is protective against HL, and lower BMI could be a significant risk factor for HL. The study results highlight the need for further research to understand the underlying molecular mechanism for this potential correlation thoroughly.

Studies showing the effect of comorbidities on cancer management and treatment are mostly reported from developed countries (Edwards et al., 2014; Panigrahi and Ambs, 2021) because of improved event reporting and disease management systems. However, studies from less developed countries are scarce, leading to a substantial gap in the understanding of comorbidities like obesity on cancer progression among diverse populations. Birhanu et al., in their original research work, assessed the prevalence of comorbidities and their associated factors in Eastern Ethiopia. The cross-sectional study took into consideration 422 cancer cases selected by a simple random sampling technique. Medical records extracted data was entered into the Epi-Data statistical software and analyzed using STATA. The study identified a lower overall prevalence of chronic comorbidities in cancer patients compared to previously reported numbers from other regions worldwide. However, a strong association between obesity and other comorbidities was identified, which influenced cancer progression. The study findings indicate that the chances of having chronic comorbidities were higher among obese cancer patients compared to lean patients. The findings further suggest that weight reduction is a measure to limit added complications and reduce cancer progression. It also provides interesting information about the lower incidence of comorbidities in cancer patients in Eastern Ethiopia, which suggests that different lifestyles and differences in genetic background can affect cancer incidence and progression differently. The observation underscores the critical need for more studies in underdeveloped and developing countries to help better understand the role of obesity and other comorbidities in cancer and other diseases.

Obesity has been reported to be associated with aggressive and high-grade prostate cancer (PCa) and increased PCa-associated deaths (Saha et al., 2023). The original research article by Kumsa et al. examined the association between neighborhood obesogenic attributes such as neighborhood socioeconomic status (nSES) with environment indices like retail-food environment index, restaurant environments index, parks, businesses, recreational facilities, and disease-associated mortality as well as incidences in prostate cancer in the Southern Community Cohort Study (SCCS). Risk analysis of PCa was further stratified by race and BMI. The study included 28,356 prostate cancer patients. Study results indicate that lower nSES, like reduced walkable areas and recreational facilities, lead to an increased prostate cancer risk and associated mortality, particularly in the African American population. The study outcomes indicate the role of obesity-associated factors in regulating PCa and highlight the importance of investigating the effect of the local environment on PCa screening and management.

Taken together, the findings from these studies clearly indicate the complex relationship between body weight and cancer progression. Further research is needed to comprehend the molecular pathways underlying obesity-associated cancer progression, identify novel biomarkers in different groups at risk, and identify new targets that can lead to the design of improved therapeutic approaches for successful clinical management of cancer. We believe that this Research Topic will serve as a valuable resource to drive further advancement in this field.
